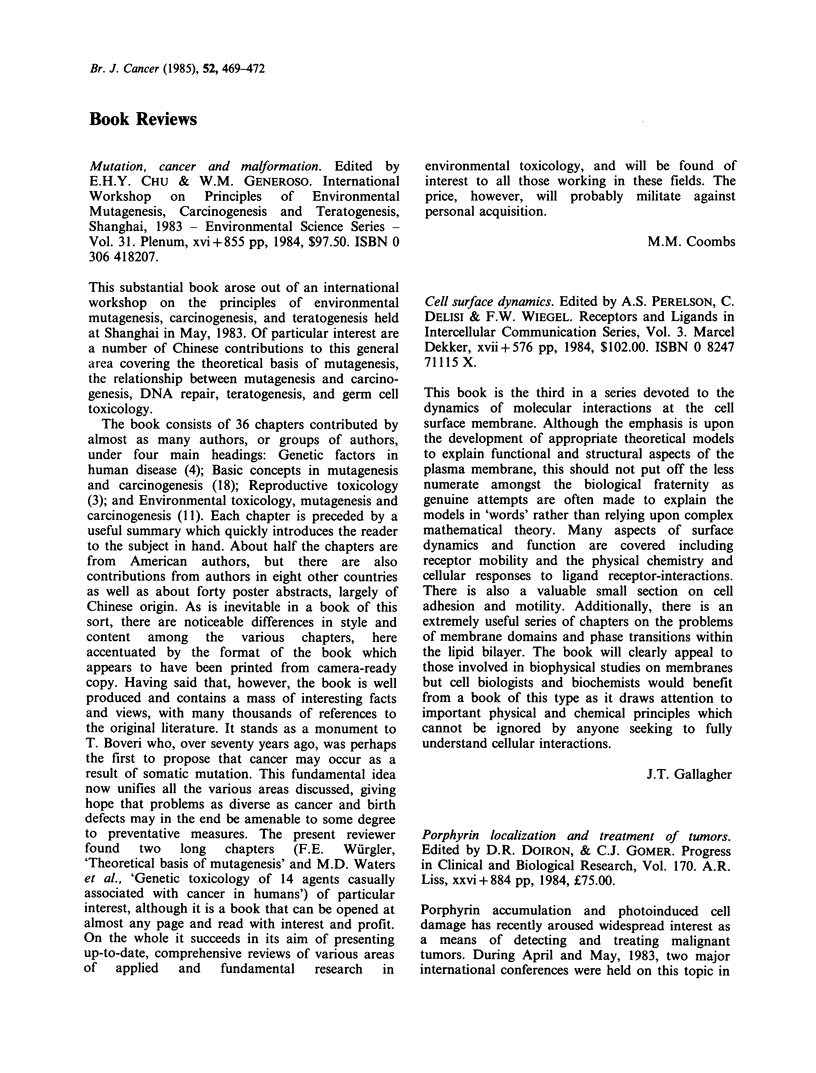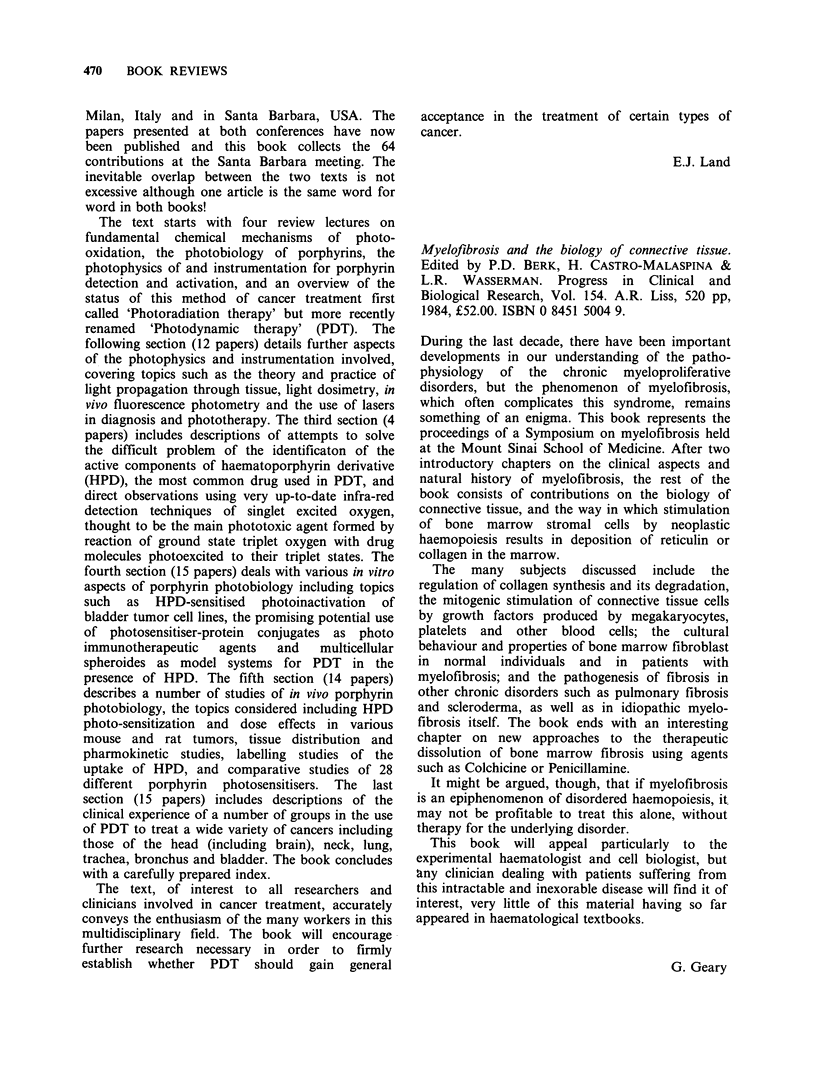# Porphyrin localization and treatment of tumors

**Published:** 1985-09

**Authors:** E.J. Land


*Porphyrin localization and treatment of tumors*. Edited by D.R. Doiron, & C.J. Gomer. Progress in Clinical and Biological Research, Vol. 170. A.R. Liss, xxvi+884 pp, 1984, £75.00.

Porphyrin accumulation and photoinduced cell damage has recently aroused widespread interest as a means of detecting and treating malignant tumors. During April and May, 1983, two major international conferences were held on this topic in

Milan, Italy and in Santa Barbara, USA. The papers presented at both conferences have now been published and this book collects the 64 contributions at the Santa Barbara meeting. The inevitable overlap between the two texts is not excessive although one article is the same word for word in both books!

The text starts with four review lectures on fundamental chemical mechanisms of photo-oxidation, the photobiology of porphyrins, the photophysics of and instrumentation for porphyrin detection and activation, and an overview of the status of this method of cancer treatment first called 'Photoradiation therapy' but more recently renamed 'Photodynamic therapy' (PDT). The following section (12 papers) details further aspects of the photophysics and instrumentation involved, covering topics such as the theory and practice of light propagation through tissue, light dosimetry, *in vivo* fluorescence photometry and the use of lasers in diagnosis and phototherapy. The third section (4 papers) includes descriptions of attempts to solve the difficult problem of the identificaton of the active components of haematoporphyrin derivative (HPD), the most common drug used in PDT, and direct observations using very up-to-date infra-red detection techniques of singlet excited oxygen, thought to be the main phototoxic agent formed by reaction of ground state triplet oxygen with drug molecules photoexcited to their triplet states. The fourth section (15 papers) deals with various *in vitro* aspects of porphyrin photobiology including topics such as HPD-sensitised photoinactivation of bladder tumor cell lines, the promising potential use of photosensitiser-protein conjugates as photo immunotherapeutic agents and multicellular spheroides as model systems for PDT in the presence of HPD. The fifth section (14 papers) describes a number of studies of *in vivo* porphyrin photobiology, the topics considered including HPD photo-sensitization and dose effects in various mouse and rat tumors, tissue distribution and pharmokinetic studies, labelling studies of the uptake of HPD, and comparative studies of 28 different porphyrin photosensitisers. The last section (15 papers) includes descriptions of the clinical experience of a number of groups in the use of PDT to treat a wide variety of cancers including those of the head (including brain), neck, lung, trachea, bronchus and bladder. The book concludes with a carefully prepared index.

The text, of interest to all researchers and clinicians involved in cancer treatment, accurately conveys the enthusiasm of the many workers in this multidisciplinary field. The book will encourage further research necessary in order to firmly establish whether PDT should gain general acceptance in the treatment of certain types of cancer.

E.J. Land